# COVID‐19 in HIV‐positive patients: A systematic review of case reports and case series

**DOI:** 10.1002/jcla.24308

**Published:** 2022-02-20

**Authors:** Mohsen Heidary, Arezoo Asadi, Negar Noorbakhsh, Shirin Dashtbin, Parisa Asadollahi, Atieh Dranbandi, Tahereh Navidifar, Roya Ghanavati

**Affiliations:** ^1^ 56941 Department of Laboratory Sciences School of Paramedical Sciences Sabzevar University of Medical Sciences Sabzevar Iran; ^2^ 56941 Cellular and Molecular Research Center Sabzevar University of Medical Sciences Sabzevar Iran; ^3^ 440827 Department of Microbiology School of Medicine Iran University of Medical Sciences Tehran Iran; ^4^ Behbahan Faculty of Medical Sciences Behbahan Iran; ^5^ Microbiology Department Faculty of Medicine Ilam University of Medical Sciences Ilam Iran; ^6^ Shoushtar Faculty of Medical Sciences Shoushtar Iran

**Keywords:** co‐infection, COVID‐19, HIV, review

## Abstract

**Introduction:**

Coronavirus disease 2019 (COVID‐19) and acquired immune deficiency syndrome (AIDS) are two viral diseases for which there are currently no definitive treatments. Nowadays, because of the health system's focus on the COVID‐19 epidemic, the control of human immunodeficiency virus (HIV) has received less attention. In this review, we will discuss the characteristics of COVID‐19 in HIV‐positive patients.

**Material and Methods:**

Using the PRISMA guideline, the databases of Scopus, PubMed, and Web of Science were searched systematically from January 1, 2019 to February 24, 2021. The following keywords were used: “Human Immunodeficiency Virus,” “acquired immune deficiency syndrome,” “HIV,” “AIDS,” “COVID‐19,” “severe acute respiratory syndrome coronavirus 2,” “novel coronavirus,” “SARS‐CoV‐2,” “nCoV disease,” “SARS2,” and “2019‐nCoV disease.”

**Results:**

Twenty‐one percent of studies were conducted in the USA (*n* = 13), 16% in China (*n* = 10), and 13% in Italy (*n* = 8), respectively. The majority of the patients were men (74.3%). Tenofovir disoproxil fumarate was used in 47.4% of patients, emtricitabine in 58.4%, and lamivudine in 34.8% to treat HIV. Symptoms of HIV patients with COVID‐19 included coughing (81.3%), fever (62.8%), and dyspnea (60%). Hydroxychloroquine (39.34%) and azithromycin (36.58%) were the common treatment options for COVID‐19. The total death rate in HIV‐positive patients with COVID‐19 was about 9%.

**Conclusion:**

In the current systematic review, we demonstrated that HIV‐positive patients co‐infected with COVID‐19 have high comorbidity of hypertension and diabetes mellitus. HIV/COVID‐19 co‐infection might have negatively influenced the HIV treatment and diagnosis, which indicates the need to regularly screen HIV patients in the COVID‐19 pandemic.

## Graphical abstract

Outcomes of patients with HIV and COVID‐19 co‐infection.

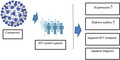

## 1 INTRODUCTION

Severe acute respiratory syndrome coronavirus 2 (SARS‐CoV‐2), a novel coronavirus, was emerged in December 2019 and COVID‐19, as its associated infection was declared as an epidemic in Wuhan, China, which turned into a pandemic in March 2020.^1^ Until August 12, 2021, more than 204 million confirmed positive cases and 4.3 million deaths have been reported worldwide.^2^

In case of new and emerging diseases, comorbidities are of special interest. It is estimated that 22% of the world's population has at least one disease that increases the risk of severe coronavirus disease 2019 (COVID‐19).^3^ According to the Centers for Disease Control and Prevention (CDC) report, people living with human immunodeficiency virus syndrome (PLWH) may be at increased risks for COVID‐19‐related complications and death.^2^ Concerns about the increased risks of severe COVID‐19 may be related to the immunosuppressive nature of HIV syndrome which makes people more susceptible to infections.^4^ There is increasing evidence that PLWH with a low CD4^+^ T‐cell count and those who do not receive antiretroviral therapy (ART) are at a higher risk of severe COVID‐19 symptoms,^5^, ^6^ even though patients with low CD4^+^ T‐cell count may be more protected against cytokine storming.^7^ One study has highlighted the possible protective effects of lymphopenia among PLWH against COVID‐19.^8^ Another study has shown that patients with low CD4^+^ T‐cell counts have a longer course of COVID‐19 and a lower antibody levels.^9^ Other risk factors such as age, sex, lung, and kidney diseases might affect the severity of COVID‐19 among PLWH.^10^

Another factor that may affect the severity of COVID‐19 is the use of ART in PLWHs. ART was proposed in 2003 as a protective factor against SARS.^11^ It can be assumed that long‐term ART may increase the risk of COVID‐19 severity,however, due to the small number of the studied cases, variable reports and insufficient data on PLWH co‐infected with COVID‐19, no certain conclusions have been obtained so far.^12^

Therefore, this systematic review was conducted to gather up the current information regarding various risk factors such as age and immune status among PLWH co‐infected with COVID‐19, as well as different antiretroviral therapies and their impacts on the disease outcome among these patients.

## 2 METHODS

The current study was carried out according to the Preferred Reporting Items for Systematic Reviews and Meta‐Analyses (PRISMA) guidelines.^13^

### 2.1 Information source and search strategies

The databases Scopus, MEDLINE (via PubMed), and Web of Science were systematically searched from January 1, 2019 to February 24, 2021 to retrieve case series and case reports published in English. The search terms included “Human Immunodeficiency Virus,” “acquired immune deficiency syndrome,” “HIV,” “AIDS,” “COVID‐19,” “severe acute respiratory syndrome coronavirus 2,” “novel coronavirus,” “SARS‐CoV‐2,” “nCoV disease,” “SARS2,” and “2019‐nCoV disease.”

### 2.2 Study selection

The case series and case reports reporting COVID‐19 among HIV‐positive patients were included. Other types of articles, including review articles, editorials, letters to editor, and guidelines, were excluded from the analysis. Moreover, duplicate publications, articles reported in languages other than English, and papers with insufficient data or available only in the abstract form were also excluded. Two different steps were taken by the authors to check the eligibility of all the potentially related articles. First, two independent authors screened the titles and abstracts and eliminated duplicate papers. Next, full text of the papers that met the inclusion criteria was reviewed.

### 2.3 Data extraction

The extracted data included the first author's name, country of the study, publication time, number of HIV/COVID‐19 co‐infected patients, HIV diagnosis methods, treatments used for the HIV infection, CD4 lymphocyte count, median duration of the HIV infection, SARS‐CoV‐2 diagnosis methods, clinical manifestations, comorbidities, therapeutic options for COVID‐19, and outcomes. Two authors independently applied the inclusion criteria to the potentially relevant articles, and disagreements between the two authors were resolved by a third author.

### 2.4 Quality assessment

The case reports/case series appraisal checklist supplied by the Joanna Briggs Institute (JBI) was used to evaluate the quality of the studies.^14^

## 3 RESULTS

**FIGURE 1:**
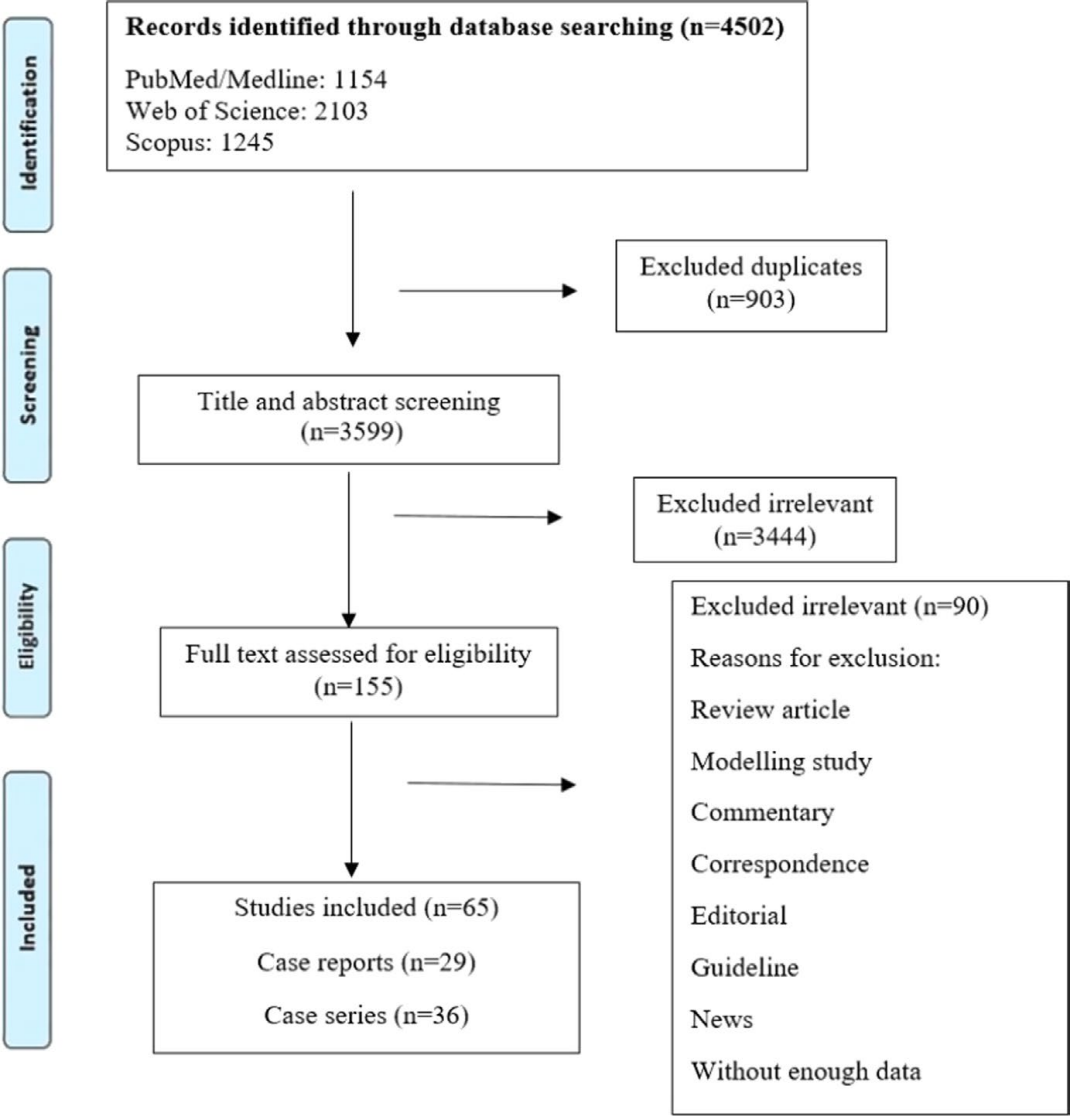
Flow diagram detailing review process and study selection

As shown in Figure 1, the initial search in the databases resulted in 4502 articles. After removing duplicates, 3599 papers remained, of which 3444 were excluded based on irrelevant titles and abstracts, followed by 155 articles retrieved for detailed full‐text evaluation. Following the full‐text evaluation, 65 articles (29 case reports and 36 case series) fulfilled the inclusion criteria and were considered for further analysis. Tables 1 and 2 show the participants’ characteristics, clinical manifestation, comorbidities, and treatment regimens obtained from the articles included in this review. Also, the demographics, clinical presentations, and the outcomes of COVID‐19 treatment among HIV‐infected individuals are summarized in Table 3. Twenty‐one percent of the studies were conducted in the USA (13 studies), 16% in China (10 studies), and 13% in Italy (8 studies). The average age of the patients was 47.9 years (ranged from 19 to 86 years). The majorities of cases were males (74.3%) and in the age range of 31–59 years (87.8%). Most of the patients had antiretroviral therapy. Tenofovir disoproxil fumarate (TDF) was used in 47.4% of the patients, followed by emtricitabine (FTC) (58.4%) and lamivudine (3TC) (34.8%) as the treatment agents for HIV‐positive participants. The most common clinical manifestations among the HIV‐positive patients with COVID‐19 were coughing (81.3%), fever (62.8%), and dyspnea (60%). The detailed clinical risk factors of individuals are shown in Table 3. Among the treatment options for COVID‐19, hydroxychloroquine (HCQ) (39.34%) and azithromycin (AZM) (36.58%) were the most commonly administered agents. Furthermore, hypertension (77%) and diabetes mellitus (20.7%) were among the most frequent comorbidities reported. The total death rate in HIV‐positive patients with COVID‐19 was about 9%.

**TABLE 1:** Characteristics of the case report studies

First author	Country	Published time	Median age (years)	Male/female	HIV treatment	Median duration of HIV infection (years)	CD4 count (Cells/mm^3^)	SARS‐CoV‐2 diagnosis method	COVID−19 treatment	Clinical manifestations	Other comorbidities	Outcomes
Alharthy (53)	Saudi Arabia	Nov 2020	40	F	LPV/r, TMP, SMX	NR	350	PCR, Chest CT	NR	Fever, Cough, Myalgias, Dyspnea	HSV‐2	Death
Giambenedetto (54)	Italy	Jun 2020	75	M	RPV, FTC, TAF, DRV, COBI	23	434	PCR, Chest CT	HCQ, AZI, PI, LMWH	Fever, Diarrhea, Cough, Dyspnea	HTN, HBV	Recovery
Bertolini (55)	Argentina	Aug 2020	43	M	TDF, FTC, DTG	NR	163	Chest CT RT‐PCR	LPV/r, HCQ	Cough, Dyspnea, Fever Night Sweats, Abdominal pain, Diarrhea	Disseminated histoplasmosis	Recovery
Basso (56)	Southern Brazil	Oct 2020	43	F	TDF, 3TC, ATV/r	21	353	Chest CT RT‐PCR	NR	Disorientation, Cough, Dyspnea, Fever	Disseminated histoplasmosis	Recovery
Bessa (57)	Brazil	Oct 2020	56	M	TDF, 3TC, EFV	NR	1,163	Chest CT RT‐PCR	CRO, CLR	Dyspnea, Asthenia	Ischemic stroke, DM	Recovery
Elhadi (58)	Libya	Aug 2020	86	F	ZDV	NR	NR	RT‐PCR Chest CT	AMX Clavulanate Pred Mucolytic syrup	Cough, Fever	T2DM	Death
Nakamoto (59)	Japan	Jan 2021	28	M	NR	NR	194	RT‐PCR Chest CT	HCQ	NR	HBV	NR
Khaba (60)	South Africa	Sep 2020	19	M	NR	NR	17	RT‐PCR Chest CT	CRO, TMP‐SMZ, HCQ, AZI, Enoxaparin	Weakness, Fatigue, Cough, SOB	NR	Death
Chiappe‐Gonzalez (61)	South America	Aug 2020	38	M	TDF‐DF, FTC, ATV/r	NR	438	RT‐PCR Chest CT	Corticosteroids	Weakness, Sore throat, Cough, Dyspnea, Diarrhea	Intra ventricular cryptococcoma	Death
Cipolat (62)	Brazil	Aug 2020	63	F	TDF, 3TC, DTG	15	426	RT‐PCR Chest CT	AMX/clavulanate, HCQ, AZI	Myalgia, Inappetence, Nausea, Abdominal pain, Diarrhea, Hyposmia, Hypogeusia, Cough, Dyspnea	NR	Recovery
Foster (63)	US	Sep 2020	40	M	LPV, RTV	NR	NR	Chest CT	HCQ, AZI	Fatigue, Cough, Dyspnea, Myalgias, Fever, Chills	NR	Recovery
Mahmood (64)	USA	Jul 2020	54	M	FTC‐TDF, Nucleotide reverse transcriptase inhibitor, DTG, Integrase Inhibitor	29	266	RT‐PCR Chest CT	HCQ, Cefuroxime	Fever, Myalgia, Cough, Dyspnea	CHD, CABG, T2DM, KSL, VAD	Recovery
Menghua (65)	China	Jul 2020	49	F	EFV, 3TC	8	224	RT‐PCR Chest CT	CRO, Interferon, Atomization, Ribavirin, Abidol	Fatigue, Fever Pharyngeal pain, Chills	NR	Recovery
Zhao (66)	China	Oct 2020	38	1 M	3TC, TDF, EFV, LPV, RTV	4	275	RT‐PCR Chest CT	NR	Fever, Muscle aches	HCV	Recovery
Baluku (67)	Uganda	Nov 2020	34	F	TDF, 3TC, EFV	5	965	RT‐PCR	HCQ, AZI, Paracetamol	Headache, Chest pain, Diarrhea, Anorexia, Fatigue	NR	Recovery
Tian (68)	China	Jul 2020	24	M	LPV/r, TDF, 3TC, EFV	2	552	RT‐PCR Chest CT	Ibuprofen, Cefotaxime, Cephalosporin	Fever	NR	Recovery
Pujari (69)	India	Jul 2020	57	M	TAF, FTC, DTG, TMP‐SMX	NR	19	RT‐PCR Chest CT	TDF, 3TC, EFV	Fever, Cough	MTB, HTN, Anemia	Recovery
Choy (70)	Singapore	Dec 2020	48	F	NR	NR	20	RT‐PCR Chest CT	Remdesivir, Corticosteroids	Cough, SOB, Diarrhea	PJP *Salmonella enteritidis*	Recovery
Muller (71)	Austria	May 2020	55	M	FTC, TDF Alafenamide, RPV	35	820	RT‐PCR Chest CT	Ampicillin/Sulbactam	Fatigue, Fever, Cough, Tachycardia	HCV, liver Transplantation, Liver cirrhosis, HCC	Recovery
Martins (72)	Portugal	Jan 2021	34	1 M	TAF	NR	77	RT‐PCR Chest CT	Cotrimoxazole, lvx Prednisolone	Fever, Cough, Bloody sputum, Anterior chest pain, Dyspnea.	Asthenia, Anorexia, Dysphagia, MSSA, PJP	Recovery
Bouare (73)	Morocco	Jul 2020	32	F	NR	NR	32	RT‐PCR Chest CT	Chloroquine, AZI, Rifampin	Fever, Cough, Headache, Myalgia	MTB	NR
d’Ettorre G (74)	Italy	July 2020	52	1F	DRV,COBI	NR	242	RT‐PCR Chest CT	HCQ	Fever	NR	Recovery
Sue (75)	China	30 Jan 2020	32	M	ZDV, 3TC, EFV	12 years	294	Chest CT	Piperacillin/TazObactam, lvx, OSE, LPV/r, Abidol	Fever, Dizziness, Cough	NR	Recovery
Chen J (76)	China	Oct 2020	24	1 M	TDF, 3TC, Favirenz	2 years	NR	RT‐PCR Chest CT	LPV/r	Fever, Cough	NR	Recovery
Tabrizian P (77)	USA	Nov 2020	57	1F	Atazanavir, FTC‐TDF, Alafenamide, RTV	24	NR	RT‐PCR Antibody testing Chest CT	NR	Dyspnea, Cough, Malaise	HCV, HCC	Recovery
Farinacci (78)	Italy	Apr 2021	59	1 M	NR	30 years	10	RT‐PCR Chest CT	HCQ, Enoxaparin	Fever, Dyspnea	NR	Death
Ji‐Yeon Kim (79)	Korea	Sep 2020	29	1 M	Genvoya, EVG, COBI, FTC, TDF	NR	555	RT‐PCR Chest CT	HCQ	Sore throat, Cough, loss of taste and smell, Chill, Myalgia, Rhinorrhea	NR	Recovery
Coleman H (80)	UK	Apr 2020	55	1 M	FTC/TDF, Disoproxil, RAL	NR	422	RT‐PCR Chest CT	NR	Fever, Cough, Hypoxia	PJP, Asthma	Recovery
Parker (81)	South Africa	Jun 2020	41	1 M	TDF, FTC, EFV	4 years	78	Chest CT	NR	Fever, Cough, Myalgia, Diarrhea, Dyspnea	PCP, TB	Recovery

Abbreviations: LPV/r, lopinavir/ritonavir; TMP, Trimethoprim; SMX, Sulfamethoxazole; TMP‐SMZ, Trimethoprim/sulfamethoxazole; RPV, Rilpivirine; FTC, Emtricitabine; TAF, Tenofovir alafenamide; DRV, darunavir; COBI, Cobicistat; TDF, tenofovir disoproxil fumarate; DTG, Dolutegravir; 3TC, Lamivudine; ATV/r, atazanavir/ritonavir; EFV, Efavirenz; ZDV, Zidovudine; LPV, Lopinavir; RTV, Ritonavir; EFV, Efavirenz; RAL, Raltegravir; HCQ, Hydroxychloroquine; AZI, Azithromycin; LMWH, low molecular weight heparin; CRO, Ceftriaxone; CLR, Clarithromycin; HSV‐2, Herpes simplex virus‐2; HTN, Hypertension; HBV, Hepatitis B; T2DM, Type 2 diabetes; CHD, Coronary heart disease; CABG, coronary artery bypass grafting; KS, *Kaposi's sarcoma*; LVAD, A left ventricular assist device; PJP or PCP, Pneumocystis jirovecii pneumonia; MSSA, Methicillin‐susceptible *Staphylococcus aureus*.

**TABLE 2:** Characteristics of the case series studies

First author	Country	Published time	Number of co‐infected patients	Median age (years)	Male/female	HIV treatment	Median duration of HIV infection (years)	CD4 count (Cells/mm^3^)	SARS‐CoV‐2 diagnosis method	COVID‐19 treatment	Clinical manifestations	Other comorbidities	Outcomes
Bartilotti‐Matos (82)	UK	Feb 2021	2	44.5	2 M	2 FTC, 2 DTG, 2 TDF, 2TMP‐SMZ	NR	122.5	PCR Chest CT	NR	1 Cough, 1 Dyspnea, 1 Diarrhea	1 HZV, 1 Weight loss, 1 Oral candidiasis, 1 Pernicious anemia	2 Recovery
Isernia (83)	France	Sep 2020	30	53.7	19 M/11F	7 ABC, 8 3TC, 3 RAL, 5 DRV 5 RTV, 12 TAF 15 FTC, 7 EVG 7 COBI, 9 DTG 7 RPV, 2 NVP 2 BIC, 1 ATV 7 TDF, 2 DOR 1 MVC, 1 ZDV	NR	500	PCR Chest CT	2 HCQ, 1 TCZ, 5 Dexamethasone	NR	11 CVD, 11 HTN, 9 DM, 7 Obesity, 5 CKD	2 Death 28 Recovery
Toombs (84)	UK	Jan 2021	3	55	2 M/1F	1 RAL, 3TC, 1 ABC, 1 TVD, 1DTG, 1 NVP	12	50/mm^3^ to 890/mm^3^	Chest CT	NR	3 Dyspnea, 1 Cough 2 Fevers, 1 anorexia, 1 Headaches	2 T2DM, 2 HTN, 1 G6PD, 1 Stroke, 1 Obesity	2 Recovery 1 Death
Li (85)	China		2	30.5	2 M	NR	NR	NR	RT‐PCR Chest CT	1 Abidor, 1 (TCZ/Abidor)	2 Intermittent, 1 Fever, 2 Chest pain, 2 Dyspnea, 2 Cough, 1 Fatigue, 1 Poor appetite, 1 Dizziness	NR	2 Recovery
Madge (86)	London	Aug 2020	18	63	14 M/4F	6 PI, 7TVD, 4ABC/3TC, 11 Integrase strand transfer Inhibitor	19.5	439	RT‐PCR Chest CT	NR	NR	3 CVD, 7 DM, 4 CKD, 8 HTN, 4 CHD, 4 COPD, 1 Breast cancer	3 Death 15 Recovery
Ridgway (30)	USA	Aug 2020	5	48	1 M/4F	1ABC, 1DTG, 1 3 TC, 2 BIC, 4 FTC, 3 TAF, 1 EVG, 1COBI, 2 RTV, 2 DRV, 1 TDF, 1 RAL	NR	257.5	RT‐PCR Chest CT	2 HCQ	5 Cough, 4 Fever, 1 SOB, 2 Headache, 2 Myalgias, 3 Diarrhea, 1 yellow sputum, 3 Diarrhea, 2 Nausea, 2 Vomiting, 1 Dehydration, 2 Chills	1 Predominantly cardiac symptoms, 1 T2DM, 1 Obstructive sleep apnea, 1 Hyperlipidemia, 2 HTN, 3 Obesity, 1 MTB,	5 Recovery
Guo (87)	China	Jun 2020	14	56	13 M/1F	7AZT, 5TDF, 11 3TC, 6 EFV, 1 RPV, 4 NVP, 1 Lpv/r, 1TAF, 1 FTC, 1 EVG/c	NR	141 to 817	RT‐PCR Chest CT	NR	10 Fever, 7 Cough, 7 Dyspnea, 11 Fatigue, 8 Blood pressure	5 HTN, 1 COPD, 1 DM, 1 Lymphoma, 1 AF, 1 Cerebral Infarction, 1 KS, 1 Bronchiectasia, 1 MTB, 1 Anemia	12 Recovery 2 Death
Sandes‐Freitas (88)	Brazil	Jan 2021	8	53.9	6 M/2F	5 DTG, 8 3TC, 3 DRV, 3 RTV, 7 ABC, 1 EFV	NR	535	NR	2 ST, 2 HCQ, 4 AZI, 3 OSE, 3 Heparin, 4 ATB, 1 Prophylactic enoxaparin	Fever, Dyspnea	6 DM, 7 HTN, 1 HCV, 1 COPD, 3 CAD, 1 PKD	3 Death 5 Recovery
Benkovic (89)	New York	Nov 2020	4	59.8	4 M	4 FTC, 4 TAF, 1 ABC, 2 DTG, 1 MVC, 1 ETR, 1 EVG, 1 COBI	23.8	794	RT‐PCR Chest CT	NR	3 Fever, 3 Fatigue, 2 Cough, 1 Diarrhea	3 Hyperlipidemia, 3 HTN, 1 HCV, 1 T2DM, 1 AF	4 Recovery
Chowdary (90)	UK	Oct 2020	2	45	1 M/1F	2 ABC, 2 3TC, 2 RAL, 2 TMP‐SMZ	14	384	RT‐PCR Chest CT	1 Doxycycline 1 Coamoxiclav	1 Myalgia, 1 Reduced appetite, 1 Weight loss, 1 Anosmia, 1 ageusia 1 Bloody diarrhea, 1 Vomiting, 1 Cough, 1 SOB, 1 Headache, 1 Malaise	2 KTR, 1 HTN, 1 RAO, 1 Asthma, 1 G6PD, 1 Monoclonal gammopathy	2 Recovery
Byrd (91)	US	Jun 2020	27	49	20 M/7F	3 ABC, 3 DTG, 2 3TC, 7 EVG/c, 2 3FTC, 22 TAF, 2 EFV, 1 TDF, 14 DTG, 1 RPV, 1 DVR/c	12	87 to 1441	RT‐PCR Chest CT	6 Remdesivir, 6 RDV	8 SOB, 2 Lethargy, 2 Fever, 4 Headache, 2 Cough, 1 Sore throat, 1 Chest pain, 1 Decreased appetite, 1 Chills, 1 Asthma	1 Dementia, 1 CVA 3 Obesity, 2 HTN, 1 DM, 1 HLD, 1 Cancer, 1 ESRD, 1 Alcoholism, 1 COPD	8 Recovery 1 Death
Zhang (92)	China	Sep 2020	2	30.5	2 M	NR	NR	NR	RT‐PCR Chest CT	2 TCZ	1 Fatigue, 1 Anorexia, 1 Dizziness, 2 Apparent, 1 Chest tightness, 2 SOB, 1 Fever, 1 Chest pain	NR	NR
Pata (93)	USA	Jul 2020	3	50.3	1F/2 M	1RTV, 1ABC, 2 DTG, 1 COBI, 1 3TC, 1 RPV, 2 darunavir, 1 BIC	NR	168	RT‐PCR Chest CT	3 HCQ, 3 AZI, 3 CRO, 1 Tamiflu	1 Diarrhea, 2 Cough, 2 SOB, 1 Abdominal pain, 1 Headaches, 1 Myalgia, 2 Fever	1 Asthma, 1 CAD, 1 HTN, 1 Hyperlipidemia, 1 Renal disease	2 Recovery 1 Death
Suwanwongse (94)	USA	Feb 2021	5	70	4 M/1F	2 FTC, 2 TAF, 1 DRV, 2 COBI, 2 DTG, 1TDF 1 RPV, 1 3TC 1 EVG	NR	37 to 1539	RT‐PCR Chest CT	4 Symptomatic 1 Convalescent plasma 1 Sarilumab trial 1HCQ	1 Rhinorrhea, 1 Abdominal Pain 1 Diarrhea, 1 Vomiting 2 Dyspnea, 2 myalgia, 1 anorexia, 3 Cough, 3 Fever, 1 Headache, 1 Nasal congestion	3 ARDS, 2 AKI, 2 Asthma, 3 HTN, 1 Alcohol abuse, 2 HCV, 2 DM 1 Hyperlipidemia 1 ESRD, 1 BPH	4 Recovery 1 Death
Rivas (95)	Panama	Aug 2020	2	41	2 M	1 TDF, 1 3TC, 1 DTG	NR	213	RT‐PCR Chest CT	2 HCQ, 1 Heparin, 1 Ceftriaxone, 1 AZI	1 Cough, 2 Dyspnea, 2 Asthenia, 2Adynamia, 1 Loss weight, 1 Fever	1 MTB	2 Recovery
Calza (28)	Italy	Jan 2021	9	56.2	7 M/2F	1 LPV/r, 4 DRV, 4 RTV, 4 COBI	21.4	258	NR	5 HCQ,3 AZI, 3 Enoxaparin	7 Fever, 7 Cough, 9 Fatigue, 7 Myalgia, 1 COPD, 7 RTI, 2 Dyspnea	1 KS, 3 PJP, 2 Interstitial Pneumonia, 6 HTN, 2 DM	9 Recovery
Farias (96)	Brazil	Aug 2020.	2	41	2 M	NR	NR	276	RT‐PCR Chest CT	2 AZI, 2 HCQ 2 Ceftriaxone	1 Fever, 1 Myalgia, 1 Headache, 2 Cough 1 Hemoptoic sputum 2 Respiratory distress	2 MTB	2 Recovery
Okoh (97)	USA	NR	27	58	15 M/12F	9 Integrase‐based regimen, 5 NNRTI 4 PI +Integrase, 3 NNRTI +Integrase, 1 PI based	NR	551	NR	7 HCQ 1 Corticosteroid 8 AZI, 8 CRO 8 Doxycycline	18 Cough, 17 Fever, 17 Dyspnea, 13 Fatigue, 9 Myalgias, 4 Diarrhea, 4 Nausea, 4 vomiting	16 HTN, 9 DM, 10 CKD, 6 Dialysis, 3 CHF, 1 CAD	27 Recovery
Harter (98)	Germany	May 2020	33	48	30 M/3F	31 NRTIs, 20 INSTI, 4 PI, 9 NNRTIs, 16 TAF, 6 TDF, 22 FTC, 9 3TC	13.9	670	RT‐PCR	6 COBI, 4 DRV, 1 RTV, 2 DOR, 6 BIC	25 Cough, 22 Fever, 7 Arthralgia, 7 Myalgia, 7 Headache, 7 Sore throat, 6 Sinusitis, 6 Anosmia	10 HTN, 6 COPD, 4 DM, 3 CVD, 5 HBV, 2 Renal insufficiency	30 Recovery 3 Death
Swaminathan (99)	USA	Nov 2020	6	64	5 M/1F	1 RPV, 1 RAL, 2 3TC, 1 ABC, 2 EFV, 2 BIC, 3 TAF, 4 FTC, 1 EVG, 1TDF	NR	765	RT‐PCR Chest CT	4 HCQ, 2 AZI 1 Corticosteroid	NR	3 Active mental health problems, 2 Active substance use(1 Tobacco, 1 Cocaine), 2 COPD, 3 DM, 1 ESRD, 2 CAD, 4 HTN, 1 PVD, 4 Hyperlipidemia	4 Recovery 2 Death
Calza (100)	Italy	Jul 2020	26	54	19 M/7F	6 PI, 5DRV/COBI, 1DRV/RTV, 16TDF/TAF, 5 RPV, 1 EFV	16.2	566	RT‐PCR Chest CT	13 HCQ, 6 AZI, 6 Enoxaparin	20 RTI, 6 Interstitial pneumonia (Most) Fever, Cough Fatigue, Myalgia, Tachypnea	14 HTN, 4 DM, 3 Obesity, 3 Asthma	26 Recovery
Biagio (101)	Italy	Nov 2020	69	53.5	50 M/19F	NR	13.5	590	RT‐PCR Chest CT	HCQ, TCZ, AZI, PI, Heparin, Corticosteroids	NR	31 HTN, 10 DM, 9 CVD	62 Recovery 7 Death
Stoeckle (21)	USA	Jul 2020	30	60.5	24 M/6F	6 PI, 4 DRV, 3 COBI, 6 DTG, 1 LPV, 4 RTV, 1 ZDV, 1 RAL, 4 3TC,7 FTC, 1 Atazanavir, 1 BIC, 1 EVG, 2 Abacavir, 2 RPV, 1 ETR, 1 Entecavir	NR	332	chest CT	HCQ, Remdesivir, Corticosteroids	17 Fever, 21 Cough, 20 Dyspnea, 10 Diarrhea, 1 Sputum, 1 Rhinorrhea, 3 Headache, 4 Myalgias, 5 Nausea, 5 vomiting 10 Diarrhea, 2 Ageusia 3 Abdominal pain, 3 Chest pain, 1 Anosmia	12 HTN, 8 DM, 1 Heart failure, 2 ESRD, 2 CAD, 4 COPD, 3 Asthma, 1 Cirrhosis, 6 HBV, 1 HCV	28 Recovery 2 Death
Przydzial (102)	USA	NR	2	57	2 M	NR	NR	63	RT‐PCR chest CT	1 AZI, 1 Piperacillin tazobactam 1 Zosyn, 1 Bactrim	Cough, Fevers, Myalgias Dyspnea Nausea/emesis	HTN, Hyperlipidemia	2 Recovery
Akyala (24)	North Central Nigeria	Sep 2020	4	29.5	4F	2 Abacavir, 2 TAF, 2 3TC, 1 FTC, 1 Alafenamide	4.7	254	RT‐PCR	1 Norfloxacin 1 γ‐globulin 1Methylprednisolone	Malaise, Cough, Fever, Headaches	1 DM, 1 Asthmatic, 1 Chronic sinusitis, 1 MTB	4 Recovery
Shekhar (27)	USA	Sep 2020	5	48.8	4 M/1F	5 FTC, 3 BIC, 1 EVG,1 RAL, 5 Tenofovir, 1 COBI	NR	603	RT‐PCR chest CT	NR	2 Chills, 1 Fatigue, 2 Fever, 2 Diarrhea, 1 SOB, 2 Cough, 1Chest discomfort, 1 Anosmia, 1 Hypogeusia, 1Abdominal pain, 1 Myalgias	2 DM, 1 CKD, 1 PAD, 1 HTN, 1 Depression, 1 Alcohol abuse	5 Recovery
Marimuthu (103)	India	Jul 2020	6	38	3 M/3F	2 ZDV, 6 3TC, 2 ZLN, 4 TDF, 3 EFV, 1 ATV/r	10.4	535	chest CT	NR	5 Fever, 2 Cough, 1 Sore throat	2 HTN, 1 EC	6 Recovery
Pinnetti (104)	Italy	Sep 2020	2	43.5	2 M	1 TDF, 1 FTC, 1 DTG, 1 EFV,	NR	127	RT‐PCR chest CT	1 HCQ	NR	2 Opportunistic infections of the (CNS), 1 HTN 1 Interstitial pneumonia	2 Recovery
Collins (105)	Georgia	Jun 2020	20	57	14 M/6F	2 NNRTIs, 4 PI, 16 INSTI,	NR	425	RT‐PCR chest CT	9 Supportive care only, 8 HCQ, 2 AZI, 1 Remdesivir	18 Cough, 13 Fever, 12 Malaise, 10 Chills, 12 SOB, 6 Diarrhea 4 Chest tightness, 6 Nausea or vomiting, 8 Myalgias, 4 Headache, 3 Sore throat, 2 Anosmia, 2 Ageusia	14 HTN, 12 Dyslipidemia 9 T2DM, 6 CVD, 6 CLD, 6 Obesity 5 CKD,3 Cancer, 8 Depression, 8 Anxiety	17 Recovery 3 Death
Gudipati (106)	USA	Oct 2020	14	56.2	12 M/2F	14 TDF, 1 DRV 1 PI/COBI	NR	612	RT‐PCR	3 Intravenous fluids, 3Corticosteroid, 14 HCQ	7 Fever, 7 SOB, 10 Cough, 4 Diarrhea, 4 loss of taste and smell	8 Obesity, 8 HTN, 6 DM, 5 CKD, 2 ESRD	11 Recovery 3 Death
Charre (26)	France	Jul 2020	77	53	52 M/25F	9 PI, 48 INSTI, 23 NNRTI, 52 TDF/TAF	15	529	RT‐PCR	NR	NR	NR	NR
Cabellos (19)	Spain	Oct 2020	63	46	56 M/7F	1 PI, 4 INSTI, 4 NNRTIs	10.8	605	RT‐PCR, IgG‐SARS‐CoV−2, IgM‐SARS‐CoV−2, Chest CT	10 Corticosteroids 13 HCQ, 4 LPV/RTV	42 Fever, 42 Cough, 31 Dyspnea, 7 Anosmia, 6 Ageusia, 14 Diarrhea, 9 Headache, 16 Weakness, 15 Myalgia/Arthralgia	12 HTN, 6 DM, 8 Overweight, 8 CVD, 3 COPD, 2 Renal chronic failure	61Recovery 2 Death
Wu Q (107)	China	Nov 2020	2	53.5	2 M	1TDF, 1 3TC, 1 EFV	6 years	NR	RT‐PCR Chest CT	1 OSE, 1 CRO 1 Moxifloxacin, 1 Tazobactam 2 Moxifloxacin 1 Ribavirin 1 Umifenovir	1 Fatigue, 2 Dyspnea, 1 Cough, 2 Myalgia, 1 SOB, 1 Sore throat, 1 Intermittent Diarrhea	1 MTB, 1 T2DM	2 Recovery
Guo W (108)	China	Aug 2020	2	NR	NR	1 SMZ/TMP	NR	NR	Chest CT IgM and IgG	NR	2 Fever, 2 Dyspnea	NR	2 Recovery
Ciccullo (109)	Italy	Jan 2020	4	19 to 43 years	4 M	3 FTC, 1 TDF, 1 RGV, 1 3TC, 3 DTG, 2 TAF	NR	516	RT‐PCR chest CT	NR	4 Fever, 3 Cough, 3 Asthaenia, 1 Sore throat	NR	4 Recovery
Cucurull‐Canosa J (110)	Spain	Jul 2020	12	62 (55–80) 51 (37–58)	7 M/5F	NR	23.6 (4–35) 21.8 (14–31)	508	RT‐PCR	7 HCQ, 7 AZI, 6 Corticosteroids	8 Fever, 3 Asthenia, 6 Respiratory symptoms, 1Gastrointestinal symptoms, 1 Headache, 4 Conjunctivitis, 3 Asymptomatic,	1 Cancer, 4 HTM, 1 DM, 1 COPD	9 Recovery 3 Death

Abbreviations: VD, Cardiovascular disease; BPH, Benign prostatic hypertrophy; CRDs, Chronic respiratory diseases; MTB, Mycobacterium tuberculosis infection; AHRF, *Acute Hypoxemic Respiratory Failure*; AKI, Acute *kidney injury*; RTIs, Respiratory tract infections; KS, *Kaposi's sarcoma*; CHF, Congestive heart failure; PJP, Pneumocystis jirovecii pneumonia; PVD, Peripheral vascular disease; CABG, coronary artery bypass grafting; HCC, Hepatocellular carcinoma; RAO, Retinal artery occlusion; EC, Esophageal candidiasis; CKD, Chronic kidney disease; CLD, Chronic lung disease; T2DM, Type 2 diabetes; HSV‐2, Herpes simplex virus‐2; HBV, Hepatitis B virus; G6PD, Glucose‐6‐phosphate dehydrogenase; CHD, Coronary heart disease; SOB, Shortness of breath; OSA, Obstructive sleep apnea; AF, Atrial fibrillation; HCV, Hepatitis C virus; COPD, Chronic obstructive pulmonary disease; *CAD*, *Coronary artery disease*; PKD, Polycystic kidney disease; KTR, kidney transplant recipients; HTN or HT, Hypertension; HLD, High‐level design; ESRD, End‐Stage Renal Disease; LVAD, A left ventricular assist device; CABG, *Coronary artery bypass* *grafting*; MSSA, Methicillin‐susceptible Staphylococcus aureus; FTC, Emtricitabine; TAF, Tenofovir alafenamide; DTG, Dolutegravir; LPV/r, lopinavir/ritonavir; TMP, Trimethoprim; SMX, Sulfamethoxazole; DRV, darunavir; HCQ, Hydroxychloroquine; TCZ, Tocilizumab; AZI, Azithromycin; TCZ, Tocilizumab; LMWH, low molecular weight heparin; CRO, Ceftriaxone; CLR, Clarithromycin; AMX, Amoxicillin; Pred, Prednisone; Ace, Acetaminophen; TMP‐SMZ, Trimethoprim/sulfamethoxazole; *HC*, Hydrocortis one; lvx, Levofloxacin; TDF, tenofovir disoproxil fumarate; 3TC, Lamivudine; RPV, Rilpivirine; COBI, Cobicistat; ATV/r, atazanavir/ritonavir; EFV, Efavirenz; RAL, Raltegravir; TVD, Truvada; NVP, Nevirapine; MVC, Maraviroc; ETR, Etravirine; EVG, Elvitegravir; LPV, Lopinavir; RTV, Ritonavir; ZDV, Zidovudine; NRTIs, nucleoside reverse transcriptase inhibitors; INSTI, integrase strand transfer inhibitors; EVG, Elvitegravir; BIC, Bictegravir.

**TABLE 3:** Summary of the case report and case series findings

	n/N (%)	No. of studies that mentioned
Sex		
Female	146/569 (25.66%)	64
Male	423/569 (74.34%)	
Age		
≤30	5/551 (0.9%)	62
30 < *n* < 60	484/551 (87.85%)	
≥60	62/551 (11.25%)	
CD		
HIV Treatment		
FTC	111/190 (58.42%)	26
DTG	42/129 (32.55%)	17
TDF	128/270 (47.41%)	31
TMP‐SMZ	6/7 (85.7%)	4
Azithromycin (AZ)	7/14 (50%)	1
ABC	28/106 (26.41%)	10
3TC	71/204 (34.8%)	24
RAL	7/76 (9.21%)	5
DRV	26/127 (20.47%)	8
RTV	21/113 (18.58%)	9
TAF	138/238 (57.98%)	15
EVG	21/126 (16.66%)	9
COBI	29/134 (21.64%)	13
RPV	21/143 (14.68%)	10
NVP	7/47 (14.89%)	3
BIC	11/79 (13.92%)	6
ATV	4/38 (10.53%)	4
TDF	114/270 (42.22%)	31
DOR	2/30 (6.66%)	1
MVC	2/34 (5.88%)	1
ZDV	7/123 (5.69%)	6
TVD	8/21 (38.09%)	2
PI	36/317 (11.35%)	9
Integrase strand transfer inhibitor	11/18 (61.11%)	1
EFV	24/98 (24.48%)	15
RPV	21/143 14.89%)	10
Lpv/r	4/25 (16%)	4
ETR	2/70 (2.85%)	2
DTG	56/129 (43.41%)	17
DVR/c	1/27 (3.70%)	1
Darunavir	2/3 (66.66%)	1
Integrase‐based regimen	17/82 (20.73%)	2
NNRTI	46/220 (20.90%)	5
NRTIs	31/33 (93.93%)	1
INSTI	88/193 (45.59%)	1
Atazanavir	2/31 (6.45%)	2
Abacavir	4/34 (11.76%)	1
Entecavir	1/30 (3.33%)	1
Tenofovir	5/5 (100%)	1
ZLN	2/6 (33.33%)	1
ATV/r	3/8 (37.5%)	3
Nucleoside and nucleotide reverse transcriptase inhibitor	1/1 (100%)	1
Integrase inhibitor	8/28 (28.57%)	2
Alafenamide	3/6 (50%)	3
Favirenz	1/1 (100%)	1
Genvoya	1/1 (100%)	1
Clinical manifestations		
Cough	192/236 (81.35%)	35
Dyspnea	95/158 (60.12%)	22
Diarrhea	52/185 (28.11%)	16
Fever	203/323 (62.84%)	44
Anorexia	4/11 (36.36%)	4
Headache	4/8 (50%)	4
Chest pain	9/63 (14.28%)	6
Fatigue	45/70 (64.28%)	13
Poor appetite	1/2 (50%)	1
Dizziness	3/5 (60%)	3
SOB	35/82 (42.68%)	11
Myalgias	26/89 (29.21%)	7
Sputum	5/40 (12.5%)	5
Nausea	18/83 (21.69%)	5
Vomiting	19/89 (21.35%)	6
Dehydration	1/5 (20%)	1
Chills	17/59 (28.81%)	6
Blood pressure	8/14 (57.14%)	1
Weight loss	2/4 (50%)	2
Anosmia	18/143 (12.59%)	6
Ageusia	11/115 (9.56%)	4
Lethargy	2/27 (7.41%)	1
Sore throat	16/94 (17.02%)	8
Abdominal pain	7/40 (17.50%)	5
Rhinorrhea	3/36 (8.33%)	3
Nasal congestion	1/5 (20%)	1
RTI	7/9 (77.77%)	1
COPD	1/9 (11.11%)	1
Arthralgia	22/96 (22.92%)	1
Myalgia	66/213 (30.98%)	20
Sinusitis	6/33 (18.18%)	1
Chest discomfort	1/5 (20%)	1
Hypogeusia	2/6 (33.33%)	2
Loss of taste and smell	5/15 (33.33%)	2
Weakness	18/65 (27.69%)	3
Respiratory symptoms	6/12 (50%)	1
Gastrointestinal symptoms	6/12 (50%)	1
Conjunctivitis	4/12 (33.33%)	1
Night Sweats	1/1 (100%)	1
Disorientation	1/1 (100%)	1
Inappetence	1/1 (100%)	1
Hyposmia	1/1 (100%)	1
Hypogeusia	2/6 (33.33%)	2
Tachycardia	1/1 (100%)	1
COVID‐19 Treatment		
HCQ	96/244 (39.34%)	26
TCZ	4/34 (11.76%)	3
Abidor	2/2 (100%)	1
ST	2/8 (25%)	1
AZI	45/123 (36.58%)	17
OSE	5/11 (45.45%)	3
Heparin	4/10 (40%)	2
ATB	4/8 (50%)	1
Enoxaparin	12/45 (26.66%)	5
Doxycycline	9/29 (31.03%)	2
Co‐amoxiclav	1/2 (50%)	1
Remdesivir	9/78 (11.54%)	4
RDV	6/27 (22.22%)	1
TCZ	4/34 (11.76%)	3
CRO	15/35 (42.86%)	6
Tamiflu	1/3 (33.33%)	1
Symptomatic	4/5 (80%)	1
Convalescent plasma	1/5 (20%)	1
Sarilumab trial	1/5 (20%)	1
Ceftriaxone	3/4 (75%)	2
Corticosteroids	23/122 (18.85%)	5
Doxycycline	9/29 (31.03%)	2
COBI	6/33 (18.18%)	1
DRV	4/33 (12.12%)	1
RTV	5/96 (5.21%)	2
DOR	2/33 (6.06%)	1
Convalescent Plasma	1/5 (20%)	1
Cotrimoxazole	1/1 (100%)	1
CD		
≤50	5/510 (0.98%)	53
50 < *n* < 200	13/510 (2.55%)	
≥200	492/510 (96.47%)	
Comorbidities		
HZV	1/2 (50%)	1
Weight loss	2/4 (50%)	2
Oral candidiasis	1/2 (50%)	1
Anemia	1/2 (50%)	1
CVD	40/233 (17.17%)	6
HTN	178/231 (77.06%)	26
DM	83/401 (20.70%)	19
Obesity	31/125 (24.8%)	7
CKD	30/114 (26.31%)	6
T2DM	15/36 (41.66%)	7
G6PD	2/5 (40%)	1
Stroke	2/4 (50%)	2
CHD	5/19 (26.32%)	2
COPD	23/211 (10.90%)	9
Cancer	6/77 (7.79%)	4
Predominantly cardiac symptoms	1/5 (20%)	1
Obstructive sleep apnea	1/5 (20%)	1
Hyperlipidemia	10/23 (43.48%)	5
MTB	9/31 (29.03%)	8
Lymphoma	1/14 (7.14%)	1
AF	2/18 (11.11%)	2
Cerebral Infarction	1/14 (7.14%)	1
KS	2/23 (8.69%)	2
Bronchiectasia	1/14 (7.14%)	1
HCV	8/50 (16%)	7
CAD	9/47 (19.14%)	5
PKD	1/8 (12.5%)	1
KTR	2/2 (100%)	1
RAO	1/2 (50%)	1
Asthma	12/71 (16.90%)	7
Monoclonal gammopathy	1/2 (50%)	1
Dementia	1/27 (3.70%)	1
CVA	1/27 (3.70%)	1
HLD	1/27 (3.70%)	1
ESRD	7/82 (8.54%)	5
Alcoholism	1/27 (3.70%)	1
Renal disease	1/3 (33.33%)	1
ARDS	3/5 (60%)	1
AKI	2/5 (40%)	1
BPH	1/5 (20%)	1
PJP	6/12 (50%)	4
Interstitial Pneumonia	3/11 (27.27%)	2
Dialysis	6/27 (22.22%)	1
CHF	3/27 (11.11%)	1
HBV	13/65 (20%)	4
HCV	7/50 (14%)	7
Active mental health problems	3/6 (50%)	1
Active substance use	2/6 (33.33%)	1
PVD	1/6 (16.66%)	1
Cirrhosis	2/31 (6.45%)	2
Depression	9/25 (36%)	2
Chronic sinusitis	1/4 (25%)	1
PAD	1/5 (20%)	1
EC	1/6 (16.66%)	1
Opportunistic infections of CNS	2/2 (100%)	1
Dyslipidemia	12/20 (60%)	1
CLD	6/20 (30%)	1
Overweight	8/63 (12.70%)	1
HSV‐2	1/1 (100%)	1
Disseminated histoplasmosis	2/2 (100%)	2
Intra ventricular cryptococcoma	1/1 (100%)	1
CABG	1/1 (100%)	1
liver transplantation	1/1 (100%)	1
HCC	2/2 (100%)	2
Asthenia	6/15 (40%)	3
Anorexia	4/11 (36.36%)	4
Dysphagia	1/1 (100%)	1
MSSA	1/1 (100%)	1
VAD	1/1 (100%)	1
PCP	1/1 (100%)	1
KSL	1/1 (100%)	1
Outcome		
Discharge	444/488 (90.98%)	61
Death	44/488 (9.02%)	

Abbreviations: VD, Cardiovascular disease; BPH, Benign prostatic hypertrophy; CRDs, Chronic respiratory diseases; MTB, Mycobacterium tuberculosis infection; AHRF, *Acute Hypoxemic Respiratory Failure*; AKI, Acute *kidney injury*; RTIs, Respiratory tract infections; KS, *Kaposi's sarcoma*; CHF, Congestive heart failure; PJP, Pneumocystis jirovecii pneumonia; PVD, Peripheral vascular disease; CABG, coronary artery bypass grafting; HCC, Hepatocellular carcinoma; RAO, Retinal artery occlusion; EC, Esophageal candidiasis; CKD, Chronic kidney disease; CLD, Chronic lung disease; T2DM, Type 2 diabetes; HSV‐2, Herpes simplex virus‐2; HBV, Hepatitis B virus; G6PD, Glucose‐6‐phosphate dehydrogenase; CHD, Coronary heart disease; SOB, Shortness of breath; OSA, Obstructive sleep apnea; AF, Atrial fibrillation; HCV, Hepatitis C virus; COPD, Chronic obstructive pulmonary disease; *CAD*, *Coronary artery disease*; PKD, Polycystic kidney disease; KTR, kidney transplant recipients; HTN or HT, Hypertension; HLD, High‐level design; ESRD, End‐Stage Renal Disease; LVAD, A left ventricular assist device; CABG, *Coronary artery bypass* *grafting*; MSSA, Methicillin‐susceptible Staphylococcus aureus; FTC, Emtricitabine; TAF, Tenofovir alafenamide; DTG, Dolutegravir; LPV/r, lopinavir/ritonavir; TMP, Trimethoprim; SMX, Sulfamethoxazole; DRV, darunavir; HCQ, Hydroxychloroquine; TCZ, Tocilizumab; AZI, Azithromycin; TCZ, Tocilizumab; LMWH, low molecular weight heparin; CRO, Ceftriaxone; CLR, Clarithromycin; AMX, Amoxicillin; Pred, Prednisone; Ace, Acetaminophen; TMP‐SMZ, Trimethoprim/sulfamethoxazole; *HC*, Hydrocortis one; lvx, Levofloxacin; TDF, tenofovir disoproxil fumarate; 3TC, Lamivudine; RPV, Rilpivirine; COBI, Cobicistat; ATV/r, atazanavir/ritonavir; EFV, Efavirenz; RAL, Raltegravir; TVD, Truvada; NVP, Nevirapine; MVC, Maraviroc; ETR, Etravirine; EVG, Elvitegravir; LPV, Lopinavir; RTV, Ritonavir; ZDV, Zidovudine; NRTIs, nucleoside reverse transcriptase inhibitors; INSTI, integrase strand transfer inhibitors; EVG, Elvitegravir; BIC, Bictegravir.

Although several extensive studies have reported the clinical characteristics of COVID‐19 and its treatment outcomes among the general population, only sparse data are available regarding the COVID‐19 status among HIV‐positive patients.^15^, ^16^ Thirty‐six case series have so far been conducted on HIV patients co‐infected with COVID‐19. In these studies, most of the participants have been on ART, have had a CD4 T‐cell count ranging from 63 to 1,441 cells/mm^3^, and have had a mild‐to‐moderate COVID‐19 disease, with symptoms like fatigue, cough, and fever.^17^, ^18^, ^19^ In 2 articles, however, it was reported that most cases had severe COVID‐19 associating with high mortality rates.^20^, ^21^ According to Table 2, COVID‐19 was diagnosed by either PCR/RT‐PCR (5 studies) (24, 26, 98, 106, and 110) or chest CT scan (3 studies) (84, 21, and 103). For the clinical diagnosis of COVID‐19, 2 studies used PCR and chest CT scans along with IgG and IgM antibody titers.^22^, ^23^ The rest of the articles included both PCR/RT‐PCR and chest CT scans and 3 not reported the criteria of diagnosis.

Table 1 summarizes the demographics, clinical presentation, and treatment outcome of HIV‐infected individuals with COVID‐19 from case reports. Twenty‐nine papers were analyzed for HIV patients with COVID‐19. In total, 65.5% of the patients surveyed were men with an age range of 19 to 75 years. Two studies have shown that old age (≥75 years), male sex, and comorbidities such as cardiac disease (CD), chronic kidney disease (CKD), and HCV infection are major risk factors of hospital admission and critical condition (17, 18). CD4 count in case reports ranged from 10 to 1,163 cells/mm^3^. About half of the cases had a CD4 count higher than 350 cells/mm^3^. In 2 cases, the COVID‐19 was diagnosed based on a Chest CT scan (75, 81). Baluku et al. confirmed the COVID‐19 by real‐time PCR (67). Tabrizian et al. used the COVID‐19 IgM and IgG serology test in addition to real‐time and chest CT scans (77). Unfortunately, despite the diagnosis of COVID‐19 and taking related treatment, 5 cases have died due to a large number of comorbidities (53, 58, 60, 61, and 78).

## 4 DISCUSSION

On March 11, 2020, the World Health Organization (WHO) labeled the new coronavirus outbreak as a pandemic. According to the latest data from Johns Hopkins University in September 12, 2021, more than 223 million COVID‐19‐positive individuals, with more than 4.6 million deaths, have been reported worldwide.^24^, ^25^ Nearly 40 million people are currently living with HIV around the world.^26^ Compromised immune responses are the main risk factors predisposing PLWH to severe forms of COVID‐19 and higher death rates.^27^ This review provides valuable information regarding various risk factors such as age and immune status among PLWH co‐infected with COVID‐19, as well as different antiretroviral therapies and their impacts on the disease outcome among these patients.

The transmission routes of HIV and SARS‐CoV‐2 are not the same; however, COVID‐19 infection can increase the transmission risk of the HIV infection. Both infections may initially present with influenza‐like symptoms such as fever, cough, and difficulty in breathing, albeit with different severities.^3^ In our study, the most prevalent clinical manifestations of COVID‐19 among PLWH were coughing (81.35%) and fever (62.84%), respectively. A longer duration of fever and lung recovery was observed in these patients as compared to COVID‐19 patients without HIV. This might be due to a delayed SARS‐CoV‐2‐specific antibody response caused by HIV infection, which can lead to a delay in the recovery of lung lesions.^28^ This delayed SARS‐CoV‐2‐specific antibody response may be due to the reduction of CD4+ T lymphocyte counts during HIV infection, as reported by numerous studies in the USA (20%), China (15.63%), and Italy (12.5%).

Sentongo et al, in a systematic review and meta‐analysis study, showed that the prevalence of HIV‐co‐infected COVID‐19 patients was significantly higher in the USA compared with Spain but was not significantly higher than that in China.^29^ Despite a high prevalence of PLWH and COVID‐19‐positive cases in Africa, only scarce data are available on the possible COVID‐19/ HIV‐co‐infected individuals,^30^ which is probably due to poor hospital records and lack of data publication in this continent.

In this study, as in other reports, most of the COVID‐19/HIV‐co‐infected patients were males (74.34%), which might be related to various behavioral, social, and biological differences between the two genders.^31^, ^32^

Among the 64 COVID‐19/HIV‐co‐infected patients with available age information, the mean age was 47.9 years which was similar to that reported by other studies^27^, ^32^ and was 2 decades younger than the mean age of the hospitalized COVID‐19 patients without HIV.

In this study, HIV‐positive patients co‐infected with COVID‐19 had high comorbidity of hypertension (77%) and diabetes mellitus (20.7%). Vizcarra et al reported a higher proportion of comorbidities among COVID‐19/ HIV‐co‐infected patients compared with COVID‐19 patients without HIV.^33^

A 9% death and 91% discharge rates have been reported among 61 COVID‐19/ HIV‐co‐infected cases for whom the information on the co‐infection outcome is available. A high discharge rate among these patients may be related to a theory that mentions PLWH are less predisposed to severe COVID‐19 infections.^34^ Ucciferri et al showed that a hyperimmune response and the subsequent cytokine storm play major roles in the pathogenicity and severity of COVID‐19 infection.^35^ Therefore, the compromised immune status of HIV‐positive individuals can explain their milder COVID‐19 symptoms, as well as a lower morbidity and mortality rates among these individuals, as compared to the HIV‐negative COVID‐19 patients.

Makoti et al noted that the respiratory load of SARS‐CoV‐2 was low in people with HIV due to the viral interference phenomenon that leads to the interruption of SARS‐CoV‐2 replication in cells already infected with HIV.^36^ In overall, there are not yet sufficient data to support or refuse any of the two hypotheses mentioned above.

The death rates among COVID‐19/HIV‐co‐infected individuals vary across different countries and regions. For example, death rates have been reported as 13.9% in the UK, 4.3% in Italy, and 3.6% in Spain, respectively.^31^ These discrepancies may be due to differences in the type of study, number of people participating in the study, characteristics of the healthcare services, and the patients’ demographic characteristics.

In this review, the majority of HIV patients with COVID‐19 co‐infection were on ART therapy. The most common antiviral drugs used in different studies for HIV‐positive patients were nucleoside reverse transcriptase inhibitors, including FTC (58.42%), TDF (47.41%), and 3TC (34.8%), respectively. These drugs have high antiviral potency, good tolerability, and low risk of severe adverse reactions associated with mitochondrial toxicity.^37^, ^38^ Hydroxychloroquine (39.24%) and azithromycin (36.58%) have been prescribed as common drugs for COVID‐19 treatment in different studies. Hydroxychloroquine (an analog of chloroquine) has been associated with a decrease in the viral load among COVID‐19 patients and has shown a comparable effect with azithromycin.^39^ There are evidences that suggest ART therapy alleviates COVID‐19 severity through immune reconstitution, but these results are not yet proven.^10^, ^40^, ^41^ In one study, most COVID‐19‐infected patients were on ART, and the median CD4 count was 752, with 96.47% having a CD4 count over 200, and only 3.53% having a CD4 count below 200. Many studies have shown an association between a low CD4 count and an increased COVID‐19 death rate among people with HIV,^42^, ^43^ which might be the reason for the 9% prevalence of death in these patients.

Co‐infection of COVID‐19 with other pathogens is one of the most important medical concerns in today's COVID‐19 pandemic days, resulting in complicated diagnosis, treatment, and disease prognosis. HIV/COVID‐19 co‐infection might have negatively influenced the HIV treatment through different means like applying social restrictions and quarantines, closure of pharmaceutical factories, and employment of health workers to care for COVID‐19 patients. Moreover, HIV/COVID‐19 co‐infection has also a serious negative impact on the 90‐90‐90 UNAIDS (the Joint United Nations Program on HIV and AIDS (UNAIDS)) strategy, which aims to end the AIDS epidemic.^44^

There are several limitations to this study, including (1) the small number of studies included (2) inclusion of only case reports and case series with small sample size and individual patient‐level data, which make it hard to generalize the data of the present study, and (3) lack of control subjects in the included studies which can insert bias into the results of the present study. The studies included in this review are observational studies which describe only a detailed description of disease occurrence in a single person or a group of individuals who all have a particular disease or condition. Therefore, these studies cannot generate information regarding rates, ratios, incidences, or prevalence of the disease conditions.

## 5 CONCLUSION

There are many reports of co‐infections associated with COVID‐19. In this review, it has been reported that most HIV/COVID‐19‐co‐infected patients, reported so far, have a high comorbidity of hypertension and diabetes mellitus and have ages above 47.9 years. HIV can increase the severity, morbidity, and mortality rates of COVID‐19 infection, but this theory has not been proven yet. On the contrary, HIV/COVID‐19 co‐infection can probably disrupt HIV treatment and diagnosis. Further studies are needed to assess the impacts of HIV infection in COVID‐19 patients.

## CONFLICT OF INTEREST

The authors declare that there is no conflict of interest.

## Data Availability

All relevant data are included in the manuscript.
